# Understanding Dendritic Cells and Their Role in Cutaneous Carcinoma and Cancer Immunotherapy

**DOI:** 10.1155/2013/624123

**Published:** 2013-03-28

**Authors:** Valerie R. Yanofsky, Hiroshi Mitsui, Diane Felsen, John A. Carucci

**Affiliations:** ^1^Department of Dermatology, NYU Langone Medical Center, New York, NY 10016, USA; ^2^Lab for Investigative Dermatology, Rockefeller University, New York, NY 10065, USA; ^3^Institute for Pediatric Urology, Weill Cornell Medical Center, New York, NY 10021, USA

## Abstract

Dendritic cells (DC) represent a diverse group of professional antigen-presenting cells that serve to link the innate and adaptive immune systems. Their capacity to initiate a robust and antigen-specific immune response has made them the ideal candidates for cancer immunotherapies. To date, the clinical impact of DC immunotherapy has been limited, which may, in part, be explained by the complex nature of DC biology. Multiple distinct subsets of DCs have been identified in the skin, where they can be broadly subcategorized into epidermal Langerhans cells (LC), myeloid-derived dermal dendritic cells (mDC) and plasmacytoid dendritic cells (pDC). Each subset is functionally unique and may activate alternate branches of the immune system. This may be relevant for the treatment of squamous cell carcinoma, where we have shown that the tumor microenvironment may preferentially suppress the activity of mDCs, while LCs remain potent stimulators of immunity. Here, we provide an in depth analysis of DC biology, with a particular focus on skin DCs and their role in cutaneous carcinoma. We further explore the current approaches to DC immunotherapy and provide evidence for the targeting of LCs as a promising new strategy in the treatment of skin cancer.

## 1. Introduction

Dendritic cells (DC) represent a small subset of immune cells that are derived from the bone marrow and are found in nearly every tissue in the human body [1]. Originally described by Steinman and Cohn in 1973 [2], these cells were found to play a critical role in linking the innate and the adaptive immune systems. This is achieved via the unique ability of DCs to sample the surrounding environment and transmit the collected information to T and B cells of the adaptive immune system [3]. DCs are considered to be professional antigen-presenting cells based on their ability to present antigen in the context of MHC class II and costimulatory molecules. They are, therefore, extremely efficient stimulators of immunity and are thought to be key players in initiating the body's immune response.

DC immunity often begins in the peripheral tissues such as the skin, where sentinel cells containing non-clonal recognition receptors will respond to specific pathogen-associated molecular patterns (PAMPs) with the secretion of protective cytokines [4]. Alternatively, peripheral DCs may ingest and process foreign antigens, followed by migration through the afferent lymphatics to the nearby lymph nodes. Antigen-derived peptides will then be loaded onto a major histocompatibility complex (MHC) for presentation to naive T cells in the lymphoid tissue [1]. Binding of T cells to the MHC-antigen complex and costimulatory molecules on the DC surface results in the activation and subsequent differentiation of T cells into effector cells capable of launching an antigen-specific response. This process is thought to be highly efficient, with only small numbers of DCs required to launch a large and successful immune attack [5]. Furthermore, nonactivated, immature DCs will also contribute to immune function through the constitutive presentation of self-antigen. Interaction with these DCs will trigger T cell deletion and the differentiation of regulatory or suppressor T cells, which effectively limits immune reactivity and generates self-tolerance. This ensures a well-controlled and targeted immune response which is limited to foreign invaders [6]. 

The potential for DCs to amplify immune function in an antigen-specific manner makes them ideal candidates for cancer immunotherapy, which attempts to eradicate tumors through the manipulation of the body's own innate immune mechanisms [7]. Mouse models have demonstrated DC tumor presentation to be an essential step in the generation of antitumor immunity; however, tumor cells themselves have been found to be poor antigen presenters [8]. Accordingly, many different DC vaccination strategies have been developed thus far, with the aim of inducing tumor-specific effector T cell responses. This may not only reduce tumor cell mass, but could also generate immunological memory, thereby preventing tumor cell relapse [9]. Such therapies may prove to be of particular importance in skin cancers, given the role of skin as a barrier to foreign invasion and the high prevalence of DCs found within the dermal and epidermal tissue [10]. Unfortunately, current approaches to DC vaccination in the treatment of human neoplasms have been largely unsuccessful. In order to better elucidate the possible mechanisms for vaccine failure, and to move forward with more effective immunotherapies, a comprehensive understanding of DC biology and its relationship to immune reactivity is required. The purpose of this paper is hence twofold: to provide an in depth analysis of DC biology, with a particular focus on skin DCs and their role in nonmelanoma skin cancers, and to highlight the various therapeutic strategies and future directions of DC immunotherapy. 

## 2. DC Biology and Plasticity 

The ability of DCs to interact with foreign antigens and initiate an immune response highlights their role as gatekeepers of the immune system. Moreover, the particular origin of a given DC, and the precise nature of a T cell interaction, can elicit a distinct pattern of differentiation [7]. For instance, DCs that reside in the lymph node typically present antigen to naive CD4+ T cells, which in turn will stimulate the production of interleukin-2 (IL-2) resulting in clonal expansion. In contrast, peripherally located DCs will often present antigen to already activated CD4+ cells, leading to the generation of effector cells [7]. 

The majority of DCs originate from CD34+ hematopoietic stem cells in the bone marrow, before entering into the circulation and depositing as immature cells in target tissues. These generally include the sites of antigen entry, such as the skin and the lining of the GI tract [11]. Alternatively, DCs may arise from monocyte precursors during times of physiologic stress. As mentioned previously, immature DCs can bind T cells and may induce immune tolerance through T cell deletion or the expansion of regulatory or suppressor T cells. They are also highly efficient at antigen recognition and processing through specialized pathogen recognition receptors (PRR) located on their cell surfaces, which respond to a variety of PAMPs [12]. Additionally, immature DCs are characterized by high levels of MHC class II molecules, which are accumulated in endosomal compartments, to facilitate antigen loading and transport to the cell surface. They also express low levels of chemokine receptors, such as CCR7, which mediate migration to the nearby lymph nodes [13]. 

Immature DCs will respond to distinct environmental signals and undergo a highly regulated maturation process resulting in activated cells capable of launching an immune response. This process is associated with the downregulation of the DC's antigen-capture capabilities, as well as increased surface expression of MHC II and other costimulatory molecules [13]. Particularly, the cross-linking of costimulatory molecule CD 40 is thought to be an essential stimulus for further DC maturation [14]. Mature DCs will also have an increased ability to secrete chemokines in order to attract naive T and B cells, and release specific cytokines to activate those cells bound to the antigen-MHC complex on their surface. Additionally, mature DCs are typically found in the lymph nodes following the acquisition of CCR7 [15]. 

While the aforementioned changes occur as necessary elements in the DC maturation process, this does not imply that maturation itself gives rise to a homogenous DC phenotype. In fact, different environmental signals produced by various microbes and nearby immune cells may contribute to the induction of unique DC phenotypes, which will ultimately shape the nature of the immune response [16]. For example, while most microbes activate DCs through PRRs, certain microbes and their associated PAMPs will actually block DC maturation. Similarly, cytokines secreted by mast cells and natural killer (NK) cells found in the tissue microenvironment may stimulate the maturation of distinct inflammatory DCs which give rise to unique T cell populations [17]. Thus depending on the cellular origins and location of an immature DC, the surrounding microenvironment, and the precise nature of a given maturation signal, multiple different DC subsets can develop which will have their own particular effect on the immune cell population and corresponding immune response.

## 3. DC Subsets in Skin

**Figure 1:**
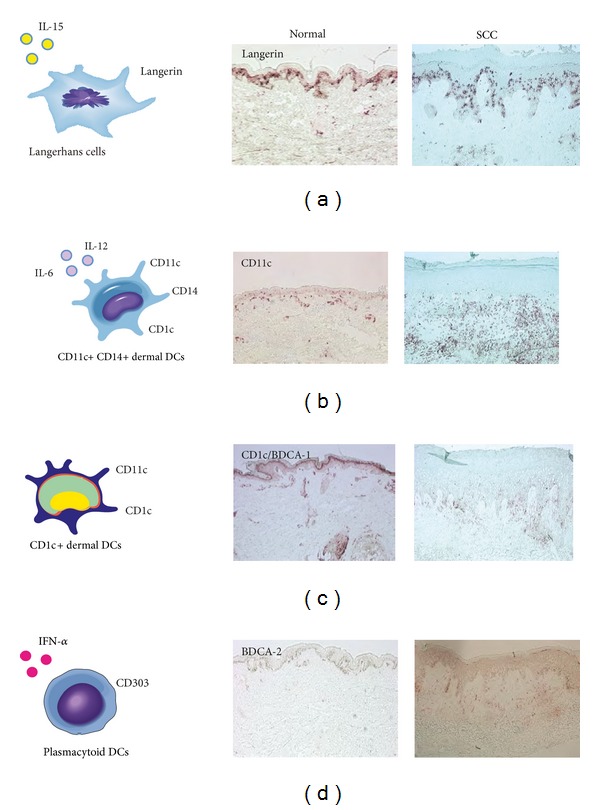
The distribution of cutaneous dendritic cell subsets in normal skin versus human squamous cell carcinoma (SCC). The human skin contains four main subsets of DCs which can be distinguished based on the differential expression of surface molecules in the steady state (left). Representative immunohistochemistry (right) demonstrating the relative distribution of DC subset markers: (a) CD207/Langerin, (b) CD11c, (c) CD1c/BDCA-1, and (d) CD303/BDCA-2.

Current research has identified four main subsets of DCs found in the human skin, which can be distinguished based on the differential expression of surface molecules in the steady state. These include the following: (1) CD1a+, CD207(Langerin)+ epidermal Langerhans cells; (2) CD11c+, CD1c(BDCA-1)+, CD14− dermal DCs; (3) CD11c+, CD1c+, CD14+ dermal DCs; and (4) CD11c−, CD303(BDCA-2)+ plasmacytoid DCs [18, 19] (Figure 1). In addition, a fifth DC subset has recently been identified in human skin, known as CD141+(BDCA-3)+, XCR1+ DCs [20, 21]. For the purpose of this paper, we will briefly discuss each subset, with a focus on the unique role it may play in shaping the immune response.

### 3.1. Langerhans Cells (LCs)

LCs are generally found in the basal and supra-basal layers of the epidermis, where they form a dense network of cells which account for approximately 2–4% of the total epidermal cell population [22, 23]. They are characterized by a unique cytoplasmic organelle known as a Birbeck granule. While the precise function of Birbeck granules remains unclear, they are thought to play a role in receptor-mediated endocytosis and the transport of cellular materials into the extracellular space [24]. Another hallmark of LCs is the expression of a specific lectin molecule, namely Langerin/CD207, which is capable of binding sugar moieties commonly found on a variety of microorganisms [10]. Although the vast majority of LCs are thought to be derived from bone marrow precursors, recent studies have identified a novel pathway by which CD14+ cells resident in the dermis will acquire LC features following treatment with TGF-*β* [25, 26]. LC differentiation may thus be somewhat dependent on the cytokine microenvironment of the skin at a given point in time. 

**Figure 2:**
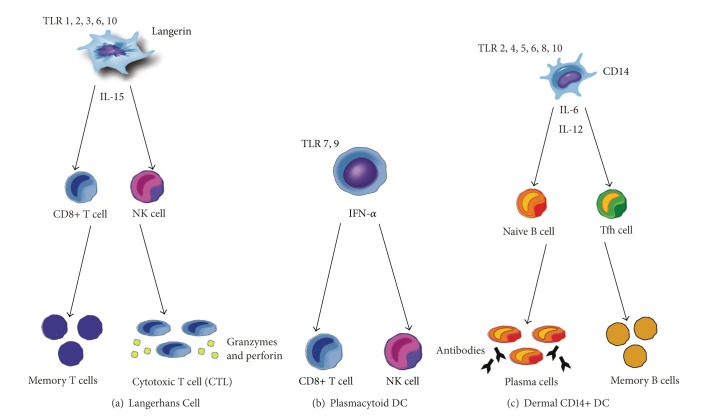
Different DC subsets have unique roles in shaping the immune response. (a) LCs are key mediators of cellular immunity and will preferentially activate CD8+ T cells and NK cells through the secretion of IL-15. LCs are also capable of cross-presenting foreign antigen, which results in a robust proliferation of naive CD8+ T cells and the generation of a highly specific cytotoxic T cell (CTL) response. This may contribute to the formation of immunologic memory. (b) PDCs respond to foreign antigen with the release of large amounts in IFN-*α*. This serves to activate a predominantly CD8+ and NK cell response. (c) Dermal CD14+ DCs are critical in the regulation of humoral immunity. When activated, they secrete IL-6 and IL-12, which promotes IgM secretion from naive B cells and the generation of mature antibody-secreting plasma cells. IL-12 will also trigger the differentiation of T follicular helper cells (Tfh) from CD4+ T cells, which may contribute to the formation of memory B cells.

LCs are thought to be key players in the initiation of cellular immunity through the stimulation of a predominantly CD8+- or NK-cell-mediated response (Figure 2(a)). LCs express a distinct set of toll-like receptors (TLRs; TLR 1, 2, 3, 6, and 10) which, when activated, result in the secretion of IL-15, a cytokine known to preferentially drive the proliferation of CD8+ T cells [27–29]. Additionally, LCs are capable of cross-presenting foreign antigens to CD8+ T cells with a greater degree of efficiency when compared to the other DC subsets, resulting in a more robust proliferation of naive CD8+ T cells [30]. This is thought to be an essential step in the initiation of a highly specific cytotoxic T cell (CTL) response, which may be critical for targeting cancer cells. In addition to their effect on CD8+ T cells, LCs may play a secondary role in the polarization of naive CD4+ T cells towards a Th2 predominant immune response through the secretion of type 2 cytokines such as IL-4, IL-5, and IL-13 [27]. 

Additionally, we have recently shown that LCs from normal human skin are capable of inducing distinct IL-22 producing CD4+ T cells (Th22) from naive CD4+ T cells *in vitro *[31]. IL-22 is thought to act mainly on epithelial cells as a key mediator of keratinocyte proliferation and epidermal hyperplasia. Additionally, we demonstrated LCs from human SCC can effectively stimulate Th1 and CD8+ T cell line expansion, which may be beneficial to antitumor immunity [32]. We are currently actively engaged in studies concerning LC-mediated T cell polarization and discovering novel means of harnessing these effects to bolster anticancer immunity. 

### 3.2. Myeloid Dendritic Cells (mDCs)

MDCs are usually found within the extracellular matrix, in the upper portion of the reticular dermis. In the past, mDCs were typically identified using an intracellular marker known as coagulation factor XIIIa. Recent studies, however, have shown this molecule to be more commonly associated with dermal macrophages [33]. Currently, mDCs are thought to be best characterized by the presence of the transmembrane integrin molecule CD11c, found at high levels on almost all human mDCs [33]. Myeloid DCs are often grossly subdivided into 2 populations based upon the differential expression of surface markers CD1c (BCDA-1) and CD14 [18, 34]. Each population is thought to represent a distinct entity with its own particular function within the greater immune environment. 

The most common mDC subtype found in normal human dermis can be identified through the use of the monoclonal antibody CD1c, also known as blood dendritic cell antigen (BDCA)-1. CD1c+ CD14− DCs are thought to be relatively immature cells that are capable of inducing only a mild T cell response [19]. Following the appropriate maturation signals, however, the immunostimulatory capacity of CD1c+ CD14− cells is greatly increased. Additionally, CD1c+ CD14− cells are thought to have a heightened sensitivity for the expression of surface receptor molecule CCR7 in response to foreign antigen detection, as well as an increased capacity for migration to nearby lymph nodes in response to the lymph node chemokine CCL19 [35]. Recently it has been suggested that in the resting state, CD1c+ CD14− DCs may in fact be tolerogenic [19].

For their part, CD1c+ CD14+ DCs are thought to be critical in the regulation of humoral immunity (Figure 2(c)). They express a novel combination of TLRs (TLR 2, 4, 5, 6, 8, and 10) which respond mainly to bacterial PAMPs, triggering the release of IL-6 and IL-12. This, in turn, serves to stimulate CD40-activated naive B cells resulting in the secretion of large amounts of IgM [30]. Additionally, only CD4+ T cells primed by CD14+ DCs are able to induce isotype switching in naive B cells. The production of mature plasma cells with antigen-specific IgG and IgA is therefore largely dependent on CD14+ DC interaction [36]. Similarly, CD4+ T cells primed by CD14+ DC will secrete high levels of CXCL13, a chemokine which promotes the homing of B cells to the follicular center [30]. 

More recently, CD14+ DCs have also been shown to induce the differentiation of a novel CD4+ T cell subtype, the IL-21 producing T follicular helper cell (Tfh). This is thought to be mediated by the release of IL-12 from CD14+ DCs, which polarizes naive T cells towards Tfh. These cells are typically found within the B cell follicle and are thought to play a role in the antigen-specific activation of naive or memory B cells. This results in the formation of unique antigen-associated germinal centers, which facilitate the transformation of B cells into high-affinity antibody secreting cells [37]. 

Additionally, we have studied the function of mDCs taken from human SCC and normal human skin and have found that SCC-associated mDCs were poor stimulators of T cell proliferation. This was true despite the fact that these cells demonstrated a mature phenotype, evidenced by the expression of cell surface molecules MHC II, CD80, CD83, and CD86 [38]. This effect may in part be explained by increased levels of the immunosuppressive cytokines TGF-*β*, IL-10 and VEGF-A, found in the tumor microenvironment. Alternatively, we have also shown that the SCC microenvironment is associated with an increased percentage of Foxp3 regulatory T cells (Tregs), which may directly inhibit the function of CD4+ and CD8+ effector T cells. Furthermore, DCs cocultured with Tregs have been shown to downregulate the expression of costimulatory molecules, which may impair their ability to stimulate T cell proliferation, thereby contributing to immune system dysfunction [39–41].

### 3.3. Plasmacytoid DCs

PDCs represent an additional population of resident dermal DCs initially identified based on their morphologic similarity to plasma cells [42]. They are often considered to be the primary foot soldiers of the innate immune system, due to their tremendous potential to produce interferon-*α* (IFN*α*) in response to viral invasion [43] (Figure 2(b)). They are characterized by the elevated expression of cell surface marker CD303 (BDCA-2), as well as a distinct set of TLRs, TLR7, and TLR9, which are specialized in the detection of viral components [44]. Furthermore, pDCs contain large stores of MHC class I molecules which enable rapid activation of CD8+ T cells to target viral antigens. PDCs are also thought to contribute secondarily to the induction of plasma cells from activated B cells, as well as the generation of immune tolerance [45, 46]. While the precise role of skin-resident pDCs has yet to be fully elucidated, recent studies have found these cells to be upregulated in the presence of cutaneous carcinomas, particularly in the juxtatumoral dermis, within 100 microns of SCC nests [38, 47]. This may reflect the importance of pDCs in the generation of a functional antitumor response, making them ideal candidates for future immunotherapy efforts. 

### 3.4. CD141+(BDCA-3)+, XCR1+ DCs

Currently, a novel subtype of human mDC has been identified in the skin, known as CD141+, XCR1+ DCs [20, 21, 48, 49]. These cells are marked by the unique coexpression of both CD141 (BDCA-3) and XCR1; however their precise phenotypic characterization remains somewhat unclear. For instance, Chu et al. have shown CD141+ DCs to be both CD11c and CD14 positive, and CD1a negative, with intermediate levels of CD1c expression [20]. In contrast, Haniffa et al. demonstrated these cells to be CD14 and CD207 negative, with low levels of CD1c, and low to intermediate expression of both CD11c and CD1a [48]. Although the exact nature of this apparent discrepancy has yet to be fully elucidated, one possible explanation includes the dramatically different mechanism of DC collection used in either study. Alternatively, these studies may indeed be describing two distinct subsets of CD141+ DCs, which may be present concurrently in the skin. 

Despite these phenotypic differences, CD141+ DCs, on a whole, are thought to possess a critical and robust ability to cross-present both self- and foreign antigen [20, 48, 49]. They have been shown to play a key role in the activation of CD25+ Tregs through both the presentation of self-antigen and the secretion of high levels of IL-10, a known immunosuppressive cytokine [20]. They may therefore be essential players in the maintenance of tissue homeostasis and the induction of immune tolerance [20]. Conversely, CD141+ DCs have also been shown to effectively cross-present soluble antigen in pathological states. This results in the generation of a powerful pro-inflammatory response [48, 49]. CD141+ DCs may therefore serve a dual role in the promotion of immune tolerance in the resting state, as well as the stimulation of immune activity in response to foreign invasion. To date, the presence and function of CD141+ DCs in the cancer microenvironment has yet to be fully explored. Further research is thus needed in order to better understand the role of CD141+ DCs in immune function, and their potential impact on cutaneous carcinomas. 

## 4. DCs and the Tumor Microenvironment 

DCs can be found in almost all human tumors, and their ability to uptake antigen and initiate an aggressive immune response makes them attractive targets for cancer immunotherapies. Moreover, while the immune system has the innate ability to recognize and attack cancer cells, tumors often evade detection by downregulating antigen presentation and impairing DC function [7]. The effective restoration of DC activity may therefore prove critical in successful tumor detection and the generation of a potent antitumor response. 

Tumors are thought to impair antigen presentation and the establishment of a tumor-specific immune response through a variety of mechanisms. For instance, tumor cells often secrete IL-6 and macrophage colony-stimulating factor (M-CSF), which may shift the differentiation of monocytes towards macrophages rather than DCs. This effectively inhibits the priming of tumor-specific T cells [50]. Furthermore, tumor cells may interfere with DC maturation through the secretion of IL-10, which results in the induction of antigen-specific anergy [51]. Tumor-derived factors have also been shown to alter the maturation pathway of DCs to produce cells which indirectly promote tumor growth. This can be accomplished through the expression of OX40 ligand on DCs, which shifts the immune response towards Th2 production. The subsequent secretion of type 2 cytokines such as IL-4 and IL-13 may actually serve to accelerate tumor growth and prevent tumor cell apoptosis [52]. 

In addition, the expression of specific tumor-generated surface receptors may prevent recognition and phagocytosis by DCs. For example, while the tumor glycoproteins carcinoembryonic antigen and mucin 1 are capable of being endocytosed by DCs, they become confined to early endosomes within cells, thus preventing processing and presentation to T cells [53]. Similarly, tumor-derived lactoferrin and CD47, which have been shown to interact with signal regulatory protein-*α* on phagocytes, will bind DCs and release inhibitory signals that will prevent phagocytosis. In fact, mouse tumor models that added a CD47-blocking antibody to the therapeutic regimen saw a marked improvement in tumor eradication, further supporting the importance of proper DC function in the generation of tumor immunity [54]. 

## 5. DCs and Cutaneous Carcinomas 

Given the rich network of DCs in the skin, these cells are often thought to be the first immune cells to encounter tumor antigens from cutaneous cancers such as squamous cell carcinoma (SCC) and basal cell carcinoma (BCC). Initiating tumor immunity may therefore be critically dependent on the proper functioning of DCs as antigen presenters, with the ability to stimulate T cell proliferation and polarization. Indeed, we and others have previously shown SCC lesions often display significantly reduced quantities of both LCs and CD11c+ dermal DCs, indicating a disruption in DC-generated immunity [38, 55]. 

We have previously studied both the phenotype and function of mDCs extracted from SCC lesions and have evaluated these cells in the context of mDCs taken from peritumoral or healthy skin. We found that tumor-associated mDCs were poor stimulators of T cell proliferation when compared to their peritumoral or healthy skin counterparts. Furthermore, we found comparable levels of the maturation markers CD83 and CD86 amongst all 3 cell types, suggesting the impairment in tumor-associated T cell activation was not the result of defective DC maturation [38]. Consistent with our findings, tumor-associated mDCs extracted from BCC lesions have also been shown to be deficient activators of the T cell response when compared to normal cutaneous mDCs [56]. While the exact mechanisms underlying this apparent discrepancy are not yet fully understood, they may in part be due to the increased expression of immunosuppressive cytokines IL-10 and TGF-*β*, which we observed in the SCC microenvironment. Additionally, we have shown the prevalence of Foxp3+ Tregs to be upregulated in cutaneous SCC lesions, which may directly inhibit the antitumor response through the suppression of CD8+ effector T cells. Furthermore, Tregs may impair DC function by inhibiting the expression of costimulatory molecules, which will interfere with DC antigen-presenting ability [39–41]. This may serve as a further factor contributing to the observed dysfunction of mature mDCs in cutaneous carcinomas.

Other unique features of the SCC microenvironment, which may play a role in the suppression of tumor-associated mDCs, include the presence of a novel CD11c+ mDC subtype known as TIP-DCs [38]. These specialized cells are characterized by the secretion of TNF and iNOS, which serve to catalyze the production of nitric oxide (NO) from L-arginine. This may result in a direct immunosuppressive effect, as elevated levels of NO have been associated with the inhibition of activated T cell proliferation [57]. Alternatively, we have previously shown mDCs in the tumor microenvironment demonstrate elevated levels of CD200 receptor expression. They may therefore be increasingly vulnerable to the effects of immunosuppressive molecules such as CD200 [58]. Lastly, we have demonstrated a prominent influx of tumor-associated macrophages in SCC lesions, which we have shown may be directly contributing to tumor growth and carcinogenesis [59]. This is mediated by the secretion of VEGF-C and MMP 9 and 11, which serve to promote tumor lymphangiogenesis and the infiltration of surrounding tissues [60–62]. Further research is currently needed in order to better understand the intricacies of the SCC microenvironment, and how they may result in the suppression of associated mDC function. 

In contrast to the diminished T cell response seen with mDCs, LCs harvested from SCC lesions have actually been shown to have an increased ability to stimulate CD4+ and CD8+ T cell proliferation *in vitro *when compared to LCs from matched, nontumor bearing skin [32]. They can also efficiently polarize T cell activity towards a predominantly Th1 response, as shown by the increased expression of IFN-*γ*. Furthermore, subsequent study revealed that nontumor LCs cultured in the presence of tumor supernatant (TSN) demonstrate an enhanced proliferation of both CD8+ and CD4+ T cells, with a shift towards a Th1 and CD8+ T cell response [32]. This suggests that the SCC microenvironment may actually serve to promote, rather than inhibit, LC activation and the initiation of the antitumor response. This stimulatory effect is appreciated despite the fact that the tumor environment is composed largely of the immunosuppressive cytokines IL-10 and TGF-*β* [38]. 

Additionally, we studied the effects of TSN on *in vitro *generated LCs and mDCs derived from CD34+ hematopoietic progenitors. We found that, similar to LCs extracted from peritumoral or healthy skin, the inclusion of TSN in the culture media effectively augmented LC-dependent T cell proliferation and Th1 polarization. However, this was not the case for mDCs, which demonstrated a markedly suppressed T cell response following treatment with TSN [32]. These results support the notion that epidermal LCs are a unique subset of DCs which, unlike other members of the DC family, may be resistant to the immunosuppressive effects of cutaneous carcinomas. They may therefore serve as critical players in the generation of SCC targeted immunotherapy. 

Given the coexistence of LCs and SCC in the human epidermis, and the enhanced ability of tumor-derived LCs to initiate type 1 immune responses *in vitro*, the question remains as to why LCs fail to prevent SCC tumor growth *in vivo. *One possible explanation for this finding is the dramatically reduced number of LCs found in both lesional and peritumoral skin [38, 55, 63]. Moreover, these cells may have impaired patterns of migration and defective mechanisms of T cell priming in the draining lymph nodes [64, 65]. The application of TSN directly to SCC lesions in mice resulted in a markedly diminished *in vivo* migration of LCs to draining lymph nodes [55, 66]. Additionally, it is worth noting that much of our knowledge concerning LC function in SCC is derived from the *ex vivo *study of migrating cells taken from preexisting tumors. Thus, the role of LCs in the tumor initiation stage is largely unknown and may be significantly different from our current observations. Accordingly, recent studies demonstrating the role of LCs in the initiation and promotion of chemically induced mouse cutaneous SCC may be of interest [67, 68].

As mentioned previously, another distinctive component of the SCC tumor microenvironment is the presence of relatively large quantities of pDCs [38]. These cells are thought to be beneficial for tumor eradication due to their inherent ability to produce large amounts of IFN-*α* in response to foreign antigen. Additionally, it has recently been shown that pDCs are capable of recognizing, processing, and cross-presenting foreign antigen to CD8+ T cells [69, 70]. Although pDCs were found to uptake reduced quantities of antigen when compared to their mDC counterparts, these findings support the notion that pDCs may still be effective mediators of the antitumor immune response [71]. Accordingly, it has been shown that the elevated amounts of pDCs are indeed associated with increased clearance of BCC lesions following treatment with imiquimod [47]. Further research is needed in order to more accurately define the role of pDCs in human cutaneous carcinomas, as well as their potential therapeutic value. 

## 6. Therapeutic Implications

**Figure 3:**
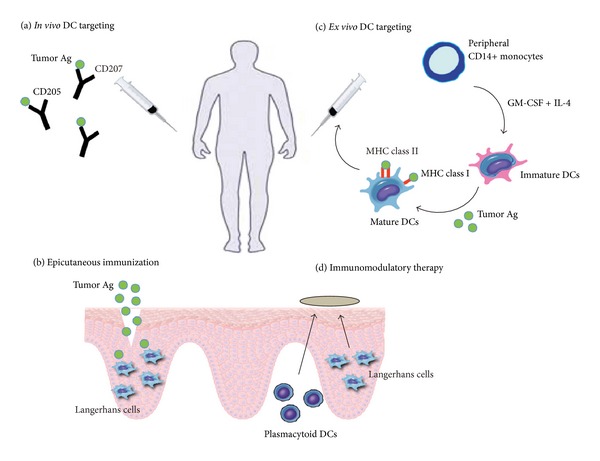
Current strategies for DC immunotherapy in the treatment of cutaneous carcinoma. (a) Direct *in vivo *DC vaccination involves the targeted delivery of tumor antigen to DCs by linking them to antibodies specific for a given DC surface molecule. Examples include CD205 for mDCs or Langerin/CD207 for LCs. (b) Epicutaneous immunization allows for the direct application of protein antigen to a disruption in the skin barrier, which will preferentially target LCs due to their predominance in the epidermis. (c) *Ex vivo *DC vaccination relies on the *in vitro* generation of immature DCs from hematopoietic progenitors or peripheral blood monocytes. DCs are then loaded with tumor antigen and reinfused into the patient. (d) Immunomodulatory therapy involves the application of topical agents that may directly regulate the immune response. Often they act as adjuvants to induce the activation and migration of LCs to nearby lymph nodes. Other effects include the TLR7/8-dependent recruitment of pDCs to the tumor region following imiquimod treatment.

The ability of DCs to link the innate and adaptive immune systems, and to generate and amplify the immune response, has made them attractive targets for tumor immunotherapy. This is particularly the case for cutaneous carcinomas, given the high prevalence of DCs in the skin, and the existence of specialized subsets with highly efficient antigen-presenting mechanisms [10]. In order for immunotherapies to be maximally effective, however, a thorough understanding of DC biology and function is required. To date, many different therapeutic approaches have been studied, with some promising initial results but limited clinical applicability. Several key DC-based therapies currently undergoing investigation for the treatment of cutaneous carcinomas include *in vivo* or epicutaneous immunization, *ex vivo* DC vaccination, and immunomodulatory therapies such as imiquimod and diphenylcyclopropenone (DPCP) administration (Figure 3). 

### 6.1. *In Vivo* and Epicutaneous Immunization

Direct *in vivo *DC vaccination involves the targeted delivery of tumor antigens to DCs through the use of chimeric proteins, which will fuse tumor antigens to antibodies specific for a given DC receptor, such as CD205 or Langerin/CD207 [72]. DC maturation signals are also commonly coadministered in order to ensure the induction of antigen-specific immunity rather than tolerance. This strategy has been shown to effectively elicit a potent CD4+ and CD8+ T cell response in mouse tumor models [73]. It also enables direct targeting of specific DC subsets by tailoring antibodies to distinct cell surface molecules. With respect to cutaneous carcinomas, targeting LC-specific molecules such as Langerin may be the key to generating the desired tumor-specific CD8+ T cell response with the induction of high-avidity CTL clones [27, 30, 32]. Additionally, direct *in vivo* delivery has the added benefit of allowing tumor-associated LCs to be activated by the local factors present in the SCC microenvironment, a step which we have shown may help bolster subsequent T cell activation [32]. 

Alternatively, *in vivo *antigen delivery can be accomplished through the direct application of protein antigen to barrier-disrupted skin, a method known as epicutaneous immunization [74]. This simple and noninvasive method will preferentially target LCs due to their predominance in the epidermis, allowing relatively easy access to topical antigen. Furthermore, disruption of the skin barrier has a pro-inflammatory effect, which serves as an adjuvant to recruit and activate LCs, and induces migration to nearby lymph nodes [75]. In mouse melanoma models, it has been shown that epicutaneous immunization is a powerful and efficient strategy for the activation of antigen-specific CD4+ and CD8+ T cell proliferation. It has also been shown to promote the induction of IFN-*γ* secreting CD8+ effector cells, which ultimately led to the successful inhibition of tumor growth [76]. Moreover, this effective antitumor response is dependent on the proper functioning of LCs, as evidenced by the markedly impaired tumor immunity seen in LC knockout mice [76]. Recent clinical trials using peptide antigen on barrier-disrupted skin in melanoma patients demonstrate the efficacious development of targeted CTL responses against tumor antigen, with the subsequent regression in tumor size and burden for a majority of patients [77]. Targeting LCs via skin immunization may therefore prove to be an important new therapy for the treatment of cutaneous carcinoma. 

### 6.2. *Ex Vivo* Generated DC Vaccines

In order to generate DC vaccines *ex vivo, *DCs must first be cultured from hematopoietic progenitor cells or peripheral blood monocytes. In the past, DCs were most commonly derived from CD34+ progenitors treated *in vitro* with granulocyte-macrophage colony-stimulating factor (GM-CSF) and tumor necrosis factor *α* (TNF-*α*) [78]. More recently, however, the preferred method of obtaining immature DCs is from peripheral blood CD14+ monocytes under tumor-free conditions, which are subsequently treated with GM-CSF and IL-4 [79]. As yet, there is no standardized method of DC preparation; thus monocytes may also be treated with alternative cytokines such as IFN-*α*, TNF-*α*, and IL-15 in combination with GM-CSF [80, 81]. Depending on the combination of cytokines used, this method allows for the preferential differentiation of distinct DC subsets, including LCs and mDCs [82]. Following the isolation of immature DCs, specific tumor antigens must be selected and loaded onto cells and then treated with the appropriate adjuvants to induce DC maturation.

Previous melanoma trials have shown that vaccination with *ex vivo *monocyte*-*derived DCs may result in the successful induction of tumor-specific CTL responses *in vivo *[83–85]. Furthermore, vaccination with mature mDCs has been associated with the generation of a detectable *in vitro *antigen-specific Th1 immune response [86]. Despite these results, the clinical impact of melanoma mDC vaccination remains relatively limited, with only a small minority of trials showing significant tumor regression [1, 87, 88]. In fact, the most common outcome seen with DC vaccination is the induction of an expanded antigen-specific immunity in the absence of any discernible clinical response [88]. 

There are several possible explanations as to why recent trials have failed to translate the immune response into an effective therapeutic outcome. First, injected DCs often fail to migrate to local lymph nodes, which may impair their ability to mount a functional immune response [89]. Additionally, proper immune function may be disrupted by the tumor microenvironment through the direct impairment of mDCs by immunosuppressive molecules such as IL-10, TGF-*β*, VEGF-A, and CD200 [38, 58]. This may be compounded by the inhibition of effector T cell function via Tregs and immune suppressive molecules such as CTLA-4 and PD-1 [38–40, 58, 90]. Lastly, the quality of DCs may be insufficient to generate a potent antitumor response *in vivo*, resulting instead in low avidity T cells which may be incapable of overcoming the immunosuppressive effects of the tumor environment [91]. In this respect, the cytokine combination used to differentiate monocytes may prove critical. Treatment with GM-CSF and IL-15 will preferentially elicit LC-type DCs, which have been shown to be more efficient in priming melanoma-antigen-specific CD8+ T cells *in vitro *than their mDC counterparts [81, 92]. This may result in the generation of powerful CD8+ effector cells, with an increased ability to target tumor antigen [92]. 

Similarly, our findings reflect the enhanced ability of LC-type DCs to stimulate CD8+ T cell proliferation *ex vivo,* in the presence of SCC-derived factors. This leads to the generation of a robust effector CD8+ T cell response, evidenced by the increased expression of IFN-*γ* [32]. LCs are therefore potent immune activators which may be resistant to inhibition from the surrounding tumor microenvironment. This occurs in direct contrast to monocyte-derived DCs, whose activity was suppressed by the tumor microenvironment. This is further reinforced by previous studies which have demonstrated that, when compared to monocyte-derived DCs, LCs are far more efficient at cross-presenting tumor antigen to CD8+ cells. This step is thought to be critical in the activation of a subsequent antigen-specific CTL response, which is necessary for successful antitumor immunity [82, 93]. Additionally, cross-presentation permits antigen loading with tumor-derived proteins rather than peptides, which allows for the generation of multiple antigenic epitopes and promotes a more powerful immune response. Furthermore, CD8+ T cells primed by LCs will show higher avidity binding and express higher levels of cytotoxic molecules such as granzymes and perforin, as compared to those primed by mDCs [88]. Accordingly, they will have a markedly improved capacity to kill target cells. This lends support to the rationale of using DC vaccines with the generation of a predominantly LC rather than mDC-like population in the treatment of cutaneous carcinoma [32].

In addition, given the powerful capacity of CD141+ DCs to cross-present foreign antigen, these cells may prove to be an important new source for the generation of *ex vivo* derived DC vaccines. Recent studies have indeed shown the successful generation of CD141+, XCR1+ DCs from induced pluripotent stem cells [94]. Furthermore, these cells were capable of effectively cross-priming cytotoxic T lymphocytes against Melan A tumor-associated antigen [94]. Although much more research is currently needed, CD141+ DCs represent attractive new targets in *ex vivo* DC vaccination therapy. 

### 6.3. Immunomodulatory Therapies

Imiquimod belongs to a class of drug known as imidazoquinolines and is an immune modulator currently approved for the treatment of a variety of cutaneous diseases such as external genital warts, actinic keratosis, and superficial BCC [95]. Although its precise mechanism of action remains unclear, imiquimod is thought to possess both antiangiogenic and proapoptotic properties and may regulate the immune response through the activation of TLR7 and TLR8 [96]. This results in the secretion of pro-inflammatory cytokines such as IFN-*α*, IL-6, and TNF-*α*. 

Imiquimod treatment is associated with the recruitment of large amounts of pDCs into the dermis and tumor microenvironment. This has been shown to result in an effective, NK-cell-dependent tumor regression in mouse melanoma models [97]. Furthermore, tumor clearance was found to be directly dependent on the corresponding pDC infiltration, with a positive correlation present between pDC numbers and the degree of tumor regression. Accordingly, imiquimod-treated pDC knockout mice were found to be incapable of successful tumor eradication [98]. The exact nature of the relationship between imiquimod treatment and pDC-dependent tumor clearance has yet to be defined; however several studies have shown that imiquimod is able to induce malignant cell apoptosis through TLR7/IFN-*α*-dependent pathways [99, 100]. Importantly, human pDCs have been found to respond to imiquimod-associated TLR7/8 stimulation with an increased expression of apoptotic factors TRAIL and FasL, as well as an increased release of cytotoxic granzyme molecules [101]. Imiquimod therapy has also been associated with elevated amounts of pDCs in human BCC lesions, resulting in more efficient tumor clearance [47]. 

An alternative mechanism that may play a role in imiquimod-mediated tumor clearance is the use of imiquimod as an adjuvant agent [102]. Multiple skin immunization studies have shown that DC adjuvants are required in order to achieve a more robust CD8+ effector T cell response [76, 102, 103]. This is thought to be due to the induction of skin inflammation, which promotes the activation and migration of LCs to nearby lymph nodes. Indeed, the application of imiquimod in conjunction with epicutaneous immunization has been associated with improved antitumor effects and more efficient tumor clearance [76]. 

Other similar immunomodulatory therapies include the use of the topical medication DPCP, which is currently approved for the treatment of alopecia areata and recalcitrant warts [104, 105]. DPCP is thought to act as a hapten or local irritant, which can induce a potent contact sensitization response, triggering the activation of epidermal LCs. Additionally, DPCP has been shown to have local immunomodulatory effects and may stimulate strong proliferative T cell responses in mice following exposure to contact allergens [106]. Recent reports have demonstrated DPCP administration alone may effectively clear skin metastases in malignant melanoma, presumably through DC activation and lymphocyte-mediated tumor destruction [107, 108]. Additionally, the adjuvant properties of DPCP have been shown to increase the efficacy of epicutaneous immunotherapy, making this drug an attractive candidate for future study [109]. 

## 7. Conclusions and Future Directions

**Figure 4:**
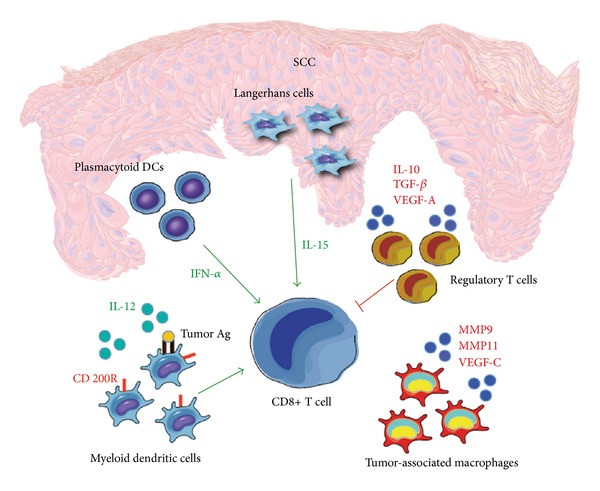
The SCC microenvironment may impact the immune response. The SCC microenvironment is dynamic and involves a complex interplay of both pro- and anti-inflammatory signals. It is associated with an elevated number of IFN-*α* secreting pDCs, and an increased capacity for LCs to stimulate CD8+ T cells. These may serve to enhance the immune response and promote tumor immunity. Conversely, we have also shown the tumor microenvironment to contain an increased number of regulatory T cells, tumor-associated macrophages, and immune suppressive molecules such as IL-10, TGF-*β*, and VEGF-A. These may be contributing to tumor growth and immune dysfunction through the suppression of mDC and CD8+ T cell activity.

For the past decade, DCs have been vigorously studied in clinical trials in order to evaluate the possibility of generating a therapeutic immune response directed against a variety of cancers. Unfortunately, while tumor-specific responses have indeed been measured in some cases, most trials have shown minimal clinical success with respect to tumor regression and overall survival rates [110]. This may, in part, be due to a limited or incomplete understanding of the role of DCs in the regulation of immunity. Moreover, predicted immune responses may be dramatically altered in the presence of intricate and complex tumor microenvironments [32, 38] (Figure 4). 

Recently, the therapeutic use of cancer vaccination has experienced a revival, owing in large part to the encouraging results seen with a number of clinical trials. Phase III trials for the treatment of metastatic prostate cancer with sipuleucel-T, a cellular product containing enriched antigen-presenting cells cultured with prostatic acid phosphatase and GM-CSF, resulted in a 4-month prolongation of the median survival time [111]. Similarly, a phase III metastatic melanoma trial, which compared peptide vaccination in combination with IL-2 therapy to IL-2 therapy alone, showed a significant improvement in overall response rate and progression-free survival in those patients who had received the vaccine [112]. 

These studies provide definitive evidence that DC immunotherapy can be exploited to yield clinically significant results. Additionally, using the skin as a therapeutic target allows for a relatively simple and minimally invasive means of investigating cancer biology. The comparative ease with which we can obtain tumor samples enables both the evaluation of clinical efficacy, and the extensive analysis of underlying tumor mechanisms and the corresponding immune reaction. Accordingly, recent years have shown considerable progress in the field of DC biology, with a greater understanding of how unique DC subsets may be interacting with tumor microenvironments to shape the immune response. This raises the possibility of developing novel therapeutic strategies, which may result in vastly improved clinical outcomes. More specifically, we believe that targeting LCs in the treatment of cutaneous carcinomas may be critical for the induction of a potent and antigen-specific CTL response, which may be resistant to the immunosuppressive effects of the local tumor environment [32]. Likewise, the addition of an appropriate adjuvant may prove beneficial in bolstering antitumor activity [76]. Although further research is needed in order to successfully translate DC biology into medicine, we believe the use of LCs in DC-based immunotherapy represents a promising new immunological approach for the treatment of cutaneous carcinomas.
